# Characterization of the mammalian RNA exonuclease 5/NEF-sp as a testis-specific nuclear 3′ → 5′ exoribonuclease

**DOI:** 10.1261/rna.060723.117

**Published:** 2017-09

**Authors:** Sara Silva, David Homolka, Ramesh S. Pillai

**Affiliations:** 1 Department of Molecular Biology, University of Geneva, CH-1211 Geneva 4, Switzerland; 2 European Molecular Biology Laboratory, Grenoble Outstation, 38042, France

**Keywords:** NEF-sp, LOC81691, Q96IC2, REXON, RNA exonuclease 5, REXO5, 2610020H08Rik

## Abstract

Ribonucleases catalyze maturation of functional RNAs or mediate degradation of cellular transcripts, activities that are critical for gene expression control. Here we identify a previously uncharacterized mammalian nuclease family member NEF-sp (RNA exonuclease 5 [REXO5] or LOC81691) as a testis-specific factor. Recombinant human NEF-sp demonstrates a divalent metal ion-dependent 3′ → 5′ exoribonuclease activity. This activity is specific to single-stranded RNA substrates and is independent of their length. The presence of a 2′-*O*-methyl modification at the 3′ end of the RNA substrate is inhibitory. Ectopically expressed NEF-sp localizes to the nucleolar/nuclear compartment in mammalian cell cultures and this is dependent on an amino-terminal nuclear localization signal. Finally, mice lacking NEF-sp are viable and display normal fertility, likely indicating overlapping functions with other nucleases. Taken together, our study provides the first biochemical and genetic exploration of the role of the NEF-sp exoribonuclease in the mammalian genome.

## INTRODUCTION

Spermatogenesis is the process by which sperm cells are produced in the male germline. Early events in this process include differentiation of embryonic primordial germ cells (PGCs) to form prospermatogonia, and later spermatogonia and spermatogonial stem cells (SSCs) (Hilscher et al. 1974). These undergo multiple rounds of amplification via mitotic divisions, which end in production of spermatocytes that enter meiosis (Bellvé et al. 1977). The spermatocytes complete meiosis to generate haploid round spermatids that enter a differentiation process called spermiogenesis which results in compaction of chromatin by removal of histones and their replacement with protamines. This is accompanied by other cellular events like formation of the acrosome that contains enzymes essential for fertilization of the egg, and formation of the flagella required for motility. Given the progressive chromatin compaction and its unavailability for transcription, some RNA products are already generated early in the spermatocytes and stored in cytoplasmic granules for translation later during spermiogenesis (Eddy 1995). Thus, RNA processing events are critical for successful completion of spermatogenesis (Dadoune et al. 2004).

Ribonucleases play an essential role in control of gene expression. All messenger RNAs (mRNAs) require ribonuclease-mediated processing to create their final 3′ ends: poly(A) tails of most mRNAs or the hairpin structure of replication-dependent histone mRNAs (Colgan and Manley 1997; Schümperli and Pillai 2004). Functional noncoding RNAs like rRNAs, tRNAs, miRNAs, piRNAs, etc. are produced as precursors that need additional ribonuclease cleavage events to produce mature RNA molecules (Allmang et al. 2000; Ghildiyal and Zamore 2009). Ribonucleases also act in important quality control steps like nonsense-mediated decay (NMD) where mistakes in transcript production that lead to premature stop codons are recognized, and such transcripts are eliminated (Mühlemann and Jensen 2012). Once these RNAs have served their purpose, removal by degradation also requires a number of ribonucleases, a step that is essential in maintenance of cellular homeostasis (Makino et al. 2013).

In our search for testis-specific nucleases, we identified the previously uncharacterized mammalian nuclease RNA exonuclease 5 (REXO5 or NEF-sp) be specifically enriched in testicular transcriptomes. We demonstrate that *Nef-sp* is exclusively expressed in mouse testes. Recombinant human NEF-sp (hNEF-sp) protein is a 3′ → 5′ exoribonuclease that acts on single-stranded RNA in a distributive manner. Modification of the RNA 3′ end is inhibitory for this activity. Ectopically expressed hNEF-sp localizes to the nucleolus in human cell cultures and this is dependent on an amino-terminal nuclear localization signal. In contrast, mouse NEF-sp is uniformly nuclear in both mouse and human cell cultures. The *Nef-sp* knockout mice are viable and fertile, indicating potential complementation by other nuclease(s). This study sheds light on the biochemical and physiological relevance of the NEF-sp exoribonuclease.

## RESULTS

### NEF-sp is a testis-specific protein

**FIGURE 1.**
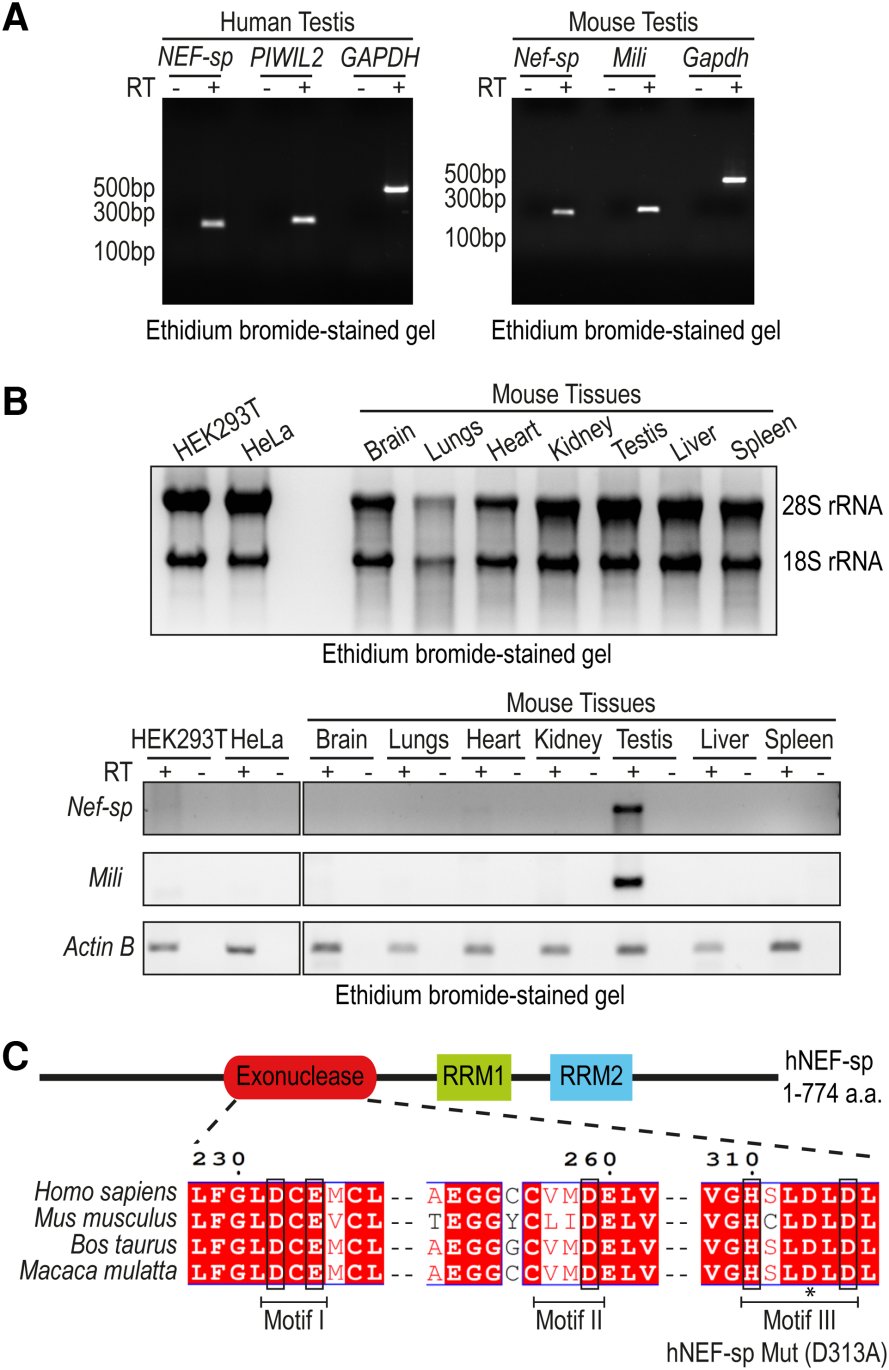
Mouse *Nef-sp* is exclusively expressed in the testes. (*A*) *Nef-sp* transcript is detected in total RNA from human and mouse testes using reverse transcription PCR (RT-PCR). Testis-specific human *PIWIL2* or mouse *Piwil2/Mili* and ubiquitously expressed *Gapdh* serve as controls. (*B*) Quality of total RNA isolated from human cell cultures (HeLa and HEK293) and different mouse tissues is indicated by ethidium bromide staining to reveal the 28S and 18S rRNAs (*top* panel). RT-PCR analysis of *Nef-sp*, *Mili*, and *Actin B* in the different RNA samples (*lower* panel). Reactions carried out without (−) and with (+) reverse transcriptase (RT) are shown. (*C*) Domain organization of human NEF-sp (hNEF-sp) showing the nuclease domain and two RNA-recognition motifs (RRMs). The conserved residues DEDDh (in boxes) distributed in three motifs, and the point mutation (D313A) introduced in motif III, are indicated.

A search for potential nucleases in testis transcriptomes led to the identification of human NEF-sp (accession no. NP_001185982) as an uncharacterized protein. It is variously known in the databases as LOC81691 or Q96IC2_REXON or 2610020H08Rik or RNA exonuclease 5 (REXO5), but in this report we will refer to it as NEF-sp. We first performed reverse transcription PCR (RT-PCR) analysis using total testes RNA (Fig. 1A). Similar to the testis-specific piRNA pathway factor *PIWIL2/Mili* (Kuramochi-Miyagawa et al. 2001), the *Nef-sp* transcript was detected in both human and mouse testes. Analysis of total RNA from various mouse tissues indicates exclusive expression of *Nef-sp* in the testes (Fig. 1B). We failed to detect the transcript in commonly used human cell cultures like HeLa or HEK293T (Fig. 1B). To study it further, we cloned complementary DNAs (cDNAs) for *Nef-sp* from human and mouse testicular total RNA. The cloned human cDNA (accession no. NP_001185982) encodes for a 774 amino acid (aa) human NEF-sp (hNEF-sp) protein that is composed of an amino-terminal nuclease domain followed by two carboxy-terminal tandem RNA-recognition motifs (RRMs) (Fig. 1C). We identified two isoforms of the mouse *Nef-sp*: a full-length long isoform encoding for a protein of 784 aa (accession no. KY853396), and a shorter carboxy-terminal splice variant encoding for a protein of 696 aa (accession no. KY853397). Both the mouse proteins have the same domain architecture, but the shorter isoform carries changes downstream from the RRM domains (see Materials and Methods).

### NEF-sp is a divalent metal ion-dependent 3′ → 5′ exonuclease

**FIGURE 2.**
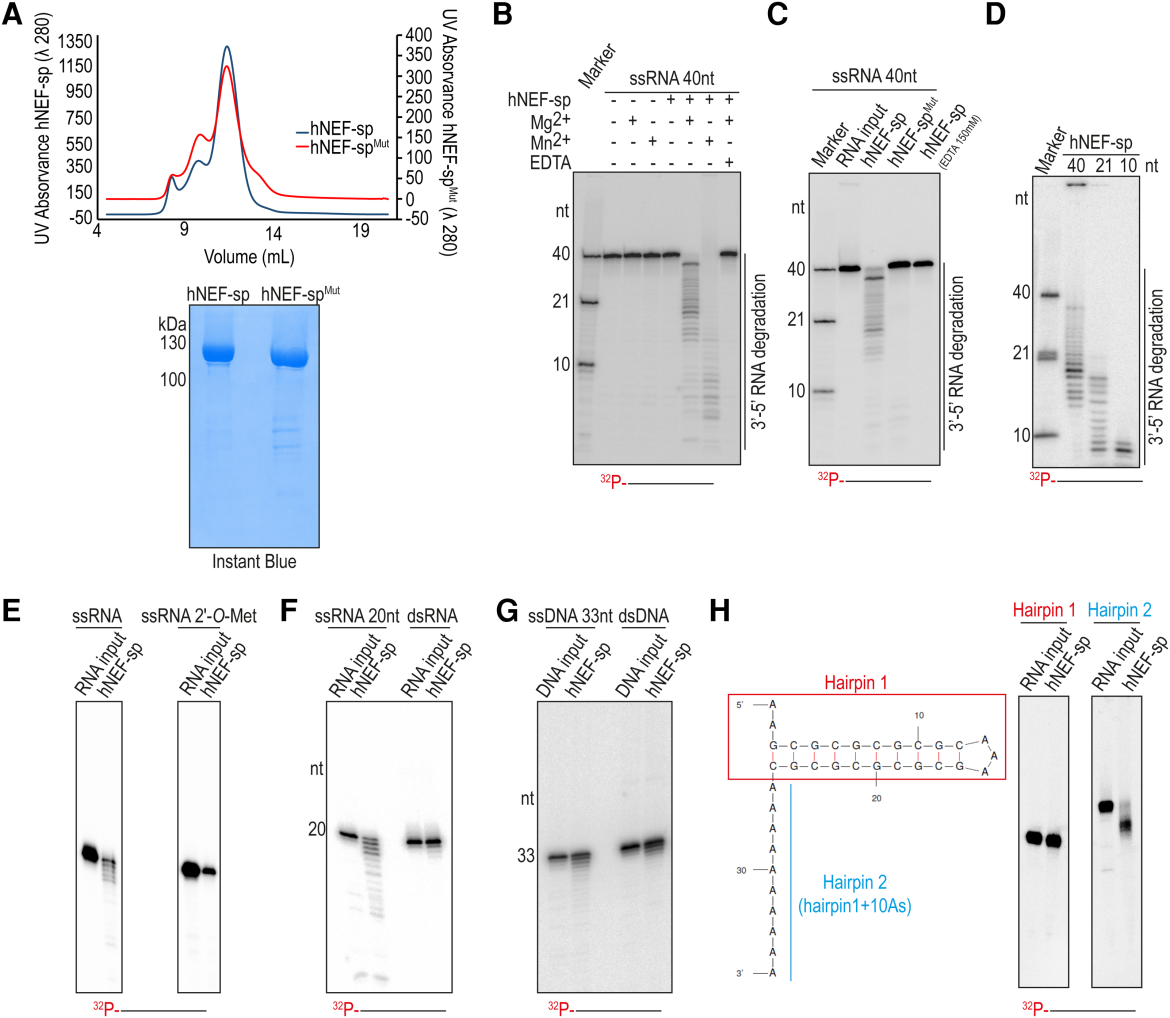
Human NEF-sp is 3′ → 5′ exoribonuclease that acts on single-stranded RNAs. (*A*) Quality of recombinant human NEF-sp wild-type and point mutant (D313A; hNEF-sp^Mut^) proteins used for nuclease assays as visualized by Instant Blue staining. The size-exclusion chromatography profiles for the proteins are shown. The identical profiles point to an absence of dramatic structural changes in the mutant protein. (*B*) Nuclease assay with a 5′-end labeled (with [^32^P-γ]ATP) 40-nt single-stranded RNA (ssRNA) and wild-type hNEF-sp protein. (*C*) Nuclease assays with wild-type and point-mutant (hNEF-sp^Mut^) hNEF-sp proteins. (*D*) Nuclease assay with ssRNA substrates of different lengths (40, 21, and 10 nt). The 10-nt RNA substrate is still digested down to at least 8–7 nt fragments. (*E*) Nuclease assay with an ssRNA carrying a 3′ terminal 2′-*O*-methyl modification. The RNA substrate is not degraded by NEF-sp (*right* panel), while the unmodified ssRNA with the same sequence is digested (*left* panel). (*F*) Reactions with 20-nt ssRNA or a 20-bp double-stranded RNA (dsRNA). Only the ssRNA is digested. (*G*) Reactions with 33-nt ssDNA or a 33-bp dsDNA indicating lack of activity to DNA substrates. (*H*) Nuclease assay using hairpin RNAs without (Hairpin 1) and with (Hairpin 2) a 3′ overhang. The structure of the hairpins as predicted by the RNA folding program Mfold is shown. Note the digestion of the single-stranded 3′ extension in Hairpin 2, but not of the double-stranded region. This experiment also shows lack of endonuclease activity on the single-stranded loop of the hairpin. Products are resolved in a 15% urea polyacrylamide gel. Markers are 5′-end labeled ssRNAs and length in nucleotides (nt) are indicated.

To examine its biochemical properties, we produced a recombinant version of the full-length human NEF-sp with an amino-terminal 6xHis-Strep-SUMO fusion tag (Fig. 2A). This tagged protein alone was incubated with a 5′-end labeled 40-nt single-stranded RNA (ssRNA) (Table 1), and the reaction products were resolved by 15% denaturing urea polyacrylamide gel electrophoresis (PAGE). This did not reveal any activity (Fig. 2B). Supplementing the reaction with divalent metal ions (Mg^2+^ or Mn^2+^) resulted in appearance of an RNA ladder, indicative of nuclease activity (Fig. 2B). We note that even when present at lower concentrations, the Mn^2+^ ion (2.5 mM) showed a stronger stimulation of the nuclease activity compared with the Mg^2+^ ion (25 mM). This is likely due to the relaxed coordination properties of Mn^2+^, compared with the stringent coordination geometry requirement of Mg^2+^ (Yang et al. 2006). The Mg^2+^-dependent exonuclease activity of human NEF-sp is completely blocked by incubation with the Mg^2+^-chelating agent EDTA (Fig. 2B). Given that the RNA substrates are 5′-end labeled, we conclude that human NEF-sp has 3′ → 5′ exonuclease activity, with the nuclease acting on all the available ssRNA substrates and degrading it in a distributive manner, giving rise to the ladder-like pattern of degradation products.

**TABLE 1.**
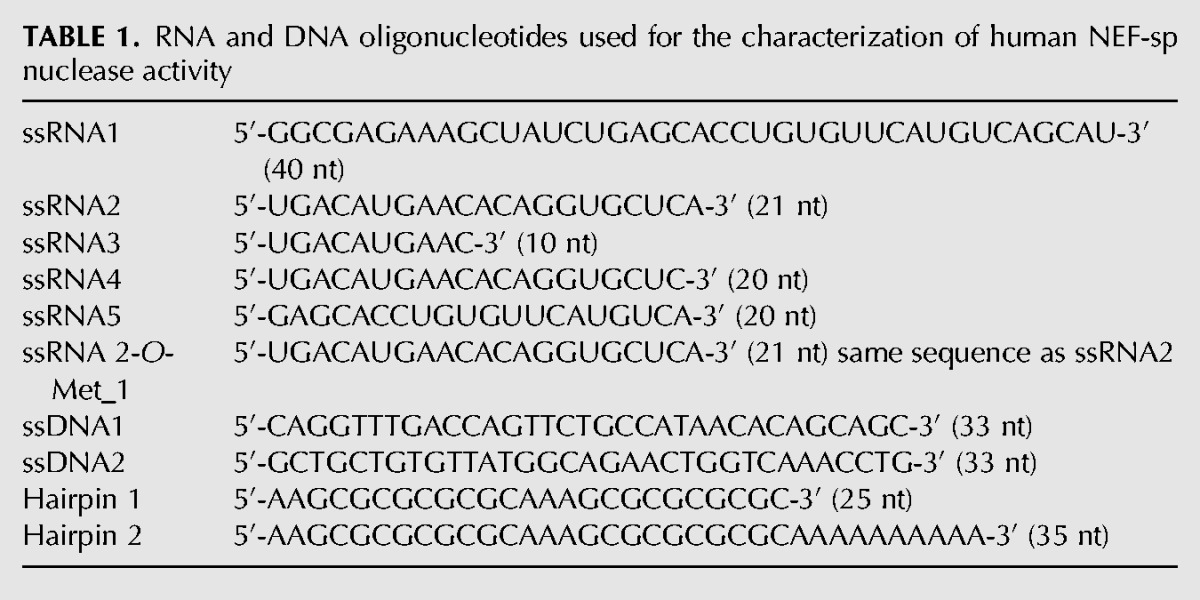
RNA and DNA oligonucleotides used for the characterization of human NEF-sp nuclease activity

Protein sequence analysis of NEF-sp identifies it as a member of the DEDD family of nucleases with the presence of four invariant acidic residues (D–E–D–D) distributed in three sequence motifs (Fig. 1C; Zuo and Deutscher 2001). The presence of a histidine in the motif III [H-x(4)-D] further identifies NEF-sp as belonging to the DEDDh subfamily. In other family members, these conserved residues are essential for coordination of two divalent metal ions required for catalytic activity. We prepared a point mutant (hNEF-sp^Mut^) version of the protein carrying a single amino acid substitution (D313A) within the motif III. The purified mutant protein behaves identical to the wild-type protein during size-exclusion chromatography, indicating lack of any gross conformational changes (Fig. 2A). Importantly, this mutation inactivates the enzyme, confirming that the nuclease activity we observe is inherent to hNEF-sp (Fig. 2C). Taken together, these results show that human NEF-sp is a divalent metal ion-dependent 3′ → 5′ exonuclease.

### Human NEF-sp acts on single-stranded RNAs

To examine the specificity of the enzyme, we incubated it with different nucleic acid substrates. First, we probed the accessibility of the catalytic pocket to single-stranded RNAs of different lengths. A 40-nt ssRNA was very efficiently used as a substrate, as already observed above. Similarly, a 21-nt and a 10-nt RNA were also used as substrates (the lane marked “marker” shows the actual undigested substrates used) (Fig. 2D). The 10-nt RNA was digested down to at least 7 nt, indicating a minimum length requirement for the ssRNA substrate to reach into the catalytic pocket of the enzyme. The 2′-*O*-methyl modification on the 3′ terminal nucleotide of RNAs is proposed to act as a stabilizing mark by protecting them from exonucleases (Ji and Chen 2012). When hNEF-sp was incubated with ssRNA carrying such a 3′ end modification, the RNA remained undigested (Fig. 2E). This shows that 2′-*O*-methyl modification of the RNA at the 3′ end hinders the activity of hNEF-sp.

Next, we examined whether double-stranded RNA (dsRNA) could be used as a substrate. When a 20-nt ssRNA was provided as a substrate, it was cleaved by the NEF-sp enzyme (Fig. 2F). However, we did not observe any activity when this sequence was preannealed with its complementary strand to make a 20-bp dsRNA duplex prior to incubation with the enzyme (Fig. 2F). This suggests that dsRNAs are not accommodated within the catalytic pocket of the enzyme. Furthermore, DNA was also a poor substrate for the enzyme in both its single-stranded and double-stranded forms (Fig. 2G). When offered structured RNAs, hNEF-sp left a 25-nt hairpin RNA (Hairpin 1) intact (Fig. 2H). However, when incubated with the same hairpin RNA, but now carrying a 3′ overhang composed of 10As (Hairpin 2), hNEF-sp was able to degrade the single-stranded region, leaving the structured part untouched (Fig. 2H). Furthermore, the above experiment also shows that hNEF-sp is not an endonuclease, as the single-stranded loop region was not cleaved. Taken together, we show that hNEF-sp is an Mg^2+^-dependent 3′ → 5′ exonuclease acting on ssRNAs and is inhibited by the presence of a 2′-*O*-methyl modification at the 3′ end of the substrate.

### Human NEF-sp is nucleolar while mouse NEF-sp is nuclear

**FIGURE 3.**
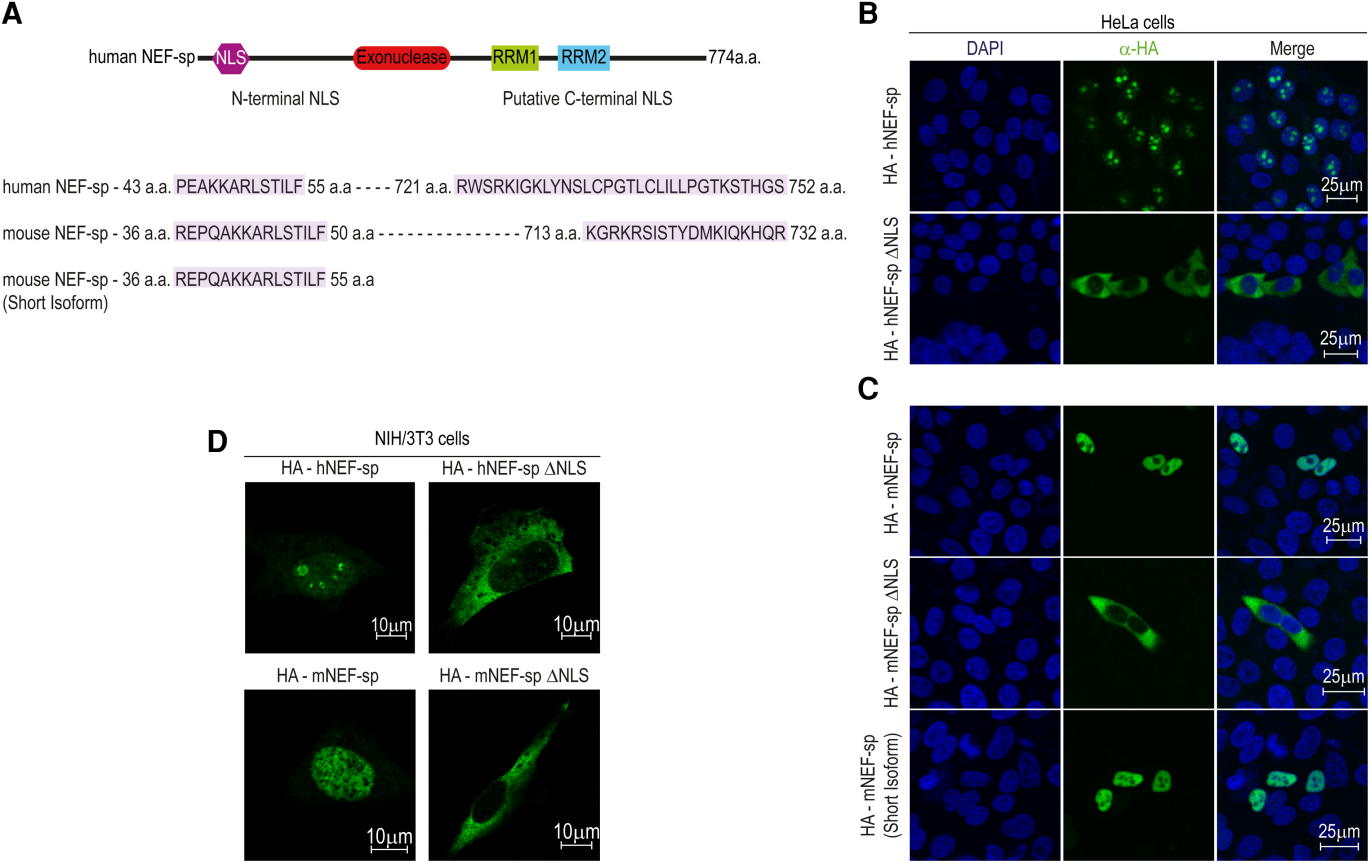
Human NEF-sp localizes to the nucleolus, while mouse NEF-sp is nuclear in human cell cultures. (*A*) Cartoon showing domain organization of hNEF-sp. The nuclear localization signal (NLS) is indicated. The predicted NLS sequences are shown *below* and amino acid (aa) boundaries are indicated. Only the amino-terminal NLS is experimentally proven to be functional. (*B*,*C*) Immunofluorescence analyses of HA-tagged proteins transiently expressed in human HeLa cell cultures. The signal from detection of the HA-tagged protein (green) and DNA with DAPI staining (blue) is shown. A merged image is provided. (*D*) Immunofluorescence detection in transfected mouse NIH3T3 cells. Scale bar in micrometers is shown.

To study its subcellular localization, we transiently expressed HA-tagged versions of NEF-sp in human HeLa cell cultures (Fig. 3A). The full-length HA-hNEF-sp protein efficiently accumulated in the nucleolus of transfected cells (Fig. 3B). Computational search for potential nuclear localization signal (NLS) sequences identified two such elements: one at the amino terminus and another at the carboxyl terminus (Fig. 3A). Of these, the amino-terminal signal is critical for nucleolar accumulation, as its deletion restricts the hNEF-sp^ΔNLS^ version to the cytoplasm (Fig. 3B). Also, a large-scale analysis of subcellular localization of human proteins revealed that hNEF-sp correctly localizes to the nucleolar compartment in a monkey cell line (Simpson et al. 2000). Curiously, although the HA-tagged mouse NEF-sp (mNEF-sp) shows nuclear localization, it was clearly excluded from the nucleoli in human HeLa cell cultures (Fig. 3C). We tested localization of mNEF-sp in mouse NIH3T3 cells and the protein was still accumulated in the nucleus while being excluded from the nucleolus, pointing to species-specific differences (Fig. 3D). Similar to the human protein, deletion of the amino-terminal NLS retains the mNEF-sp^ΔNLS^ version in the cytoplasm. The short isoform of mNEF-sp which lacks the predicted carboxy-terminal NLS (Fig. 3A) was still efficiently imported into the nucleus (Fig. 3C). Thus we conclude that the amino-terminal NLS is necessary and sufficient for nuclear/nucleolar import of NEF-sp. Our attempts to examine endogenous mNEF-sp in mouse testes with anti-mNEF-sp antibodies was unsuccessful. Taken together, we propose that NEF-sp is an exoribonuclease that may function in the nuclear/nucleolar compartment.

### Analysis of *Nef-sp* knockout mouse

**FIGURE 4.**
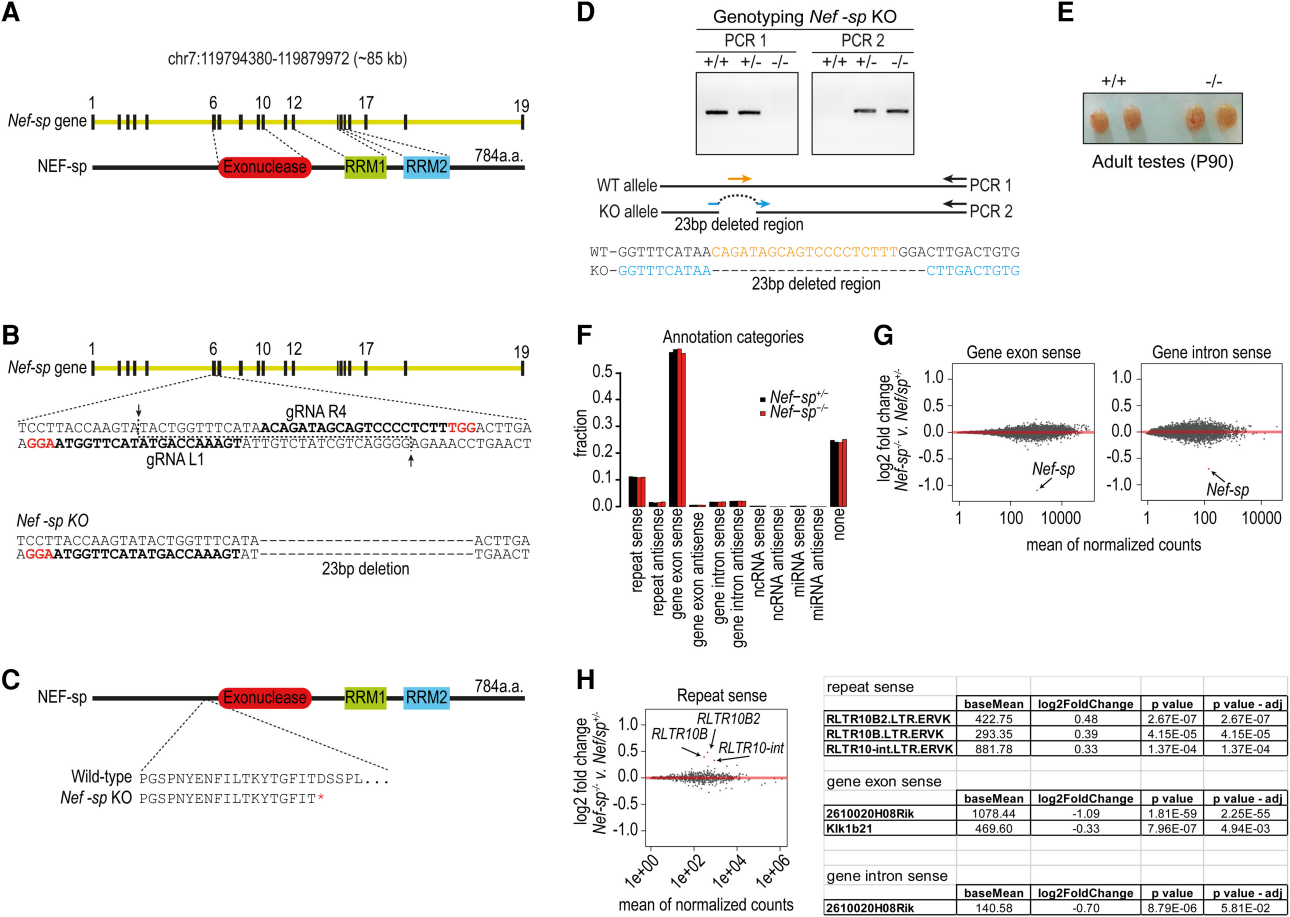
*Nef-sp* knockout mice are viable and fertile. (*A*) Gene organization of mouse *Nef-sp* and mNEF-sp protein domain architecture. (*B*) Guide RNAs (gRNAs) used for generation of a 23-bp deletion in the mouse *Nef-sp* gene locus of single-cell embryos. (*C*) Premature translation stop codon (D220*) introduced by the deletion leading to a truncation of the protein before the nuclease domain. (*D*) Genotyping of the wild-type, *Nef-sp*^+/−^ and *Nef-sp*^−/−^ mice. Cartoon showing the set of primers (represented by arrows) used to distinguish the wild-type allele from the knockout allele. See Materials and Methods for details. (*E*) Representative mouse testes from wild-type and mutant Nef-sp animals showing normal testes size. Mutant mice show normal fertility. (*F*) Analysis of the total testicular transcriptome from heterozygous and homozygous *Nef-sp* knockout mutant mice. Overall genome annotations of reads do not show any changes. (*G*) Comparison of read counts of sense-oriented gene exonic and intronic reads reveal down-regulation of *Nef-sp* transcript, as expected. No other significant change in transcripts is noted. (*H*) Analysis of sense-oriented repeat reads shows an up-regulation of the indicated repeat elements. Table showing fold-changes in read counts of sense-oriented repeat elements and *Nef-sp* transcript.

To study the in vivo role of NEF-sp, we introduced a deletion in the *Nef-sp* gene locus in single-cell mouse embryos using the RNA-guided Cas9 genome-editing enzyme (Fig. 4A,B). The generated mutant mice have a 23 bp deletion in the coding sequence, resulting in a premature stop codon (D220*) upstream of the nuclease domain (Fig. 4C,D). We will refer to this as the *Nef-sp* knockout allele (*Nef-sp*^−^). Homozygous *Nef-sp*^−/−^ males displayed normal testes sizes when compared to wild-type animals (Fig. 4E). We report that homozygous *Nef-sp*^−/−^ animals of both sexes display normal fertility and viability. Deep sequencing of testicular total RNAs from wild-type and homozygous knockout mice did not reveal any dramatic changes in transcripts belonging to the different annotation categories (Fig. 4F). Closer examination of genic cellular mRNAs showed unchanged levels, except for a dramatic reduction in *Nef-sp* transcript levels in the knockout mutant as expected from the deletion (Fig. 4G). Examination of repeat-associated transcripts reveals a modest increase in RLTR10B, RLTR10B2, and RLTR10-int transposon sequences (Fig. 4H). We did not observe any other changes in these sequenced transcriptomes. Taken together with the fact that the knockout mutants are fertile, this indicates that loss of NEF-sp is tolerated, perhaps via complementation by an unknown nuclease(s).

## DISCUSSION

Here we examined the biochemical properties of the uncharacterized mammalian nuclease family member NEF-sp. We show that *Nef-sp* transcripts are detected exclusively in mouse testes. Our efforts to detect the endogenous protein in mouse testes extracts and visualize it in testes sections failed. Since our rabbit polyclonal antibodies detect transiently expressed HA-mNEF-sp in human cell cultures (data not shown), we conclude that perhaps abundance of the endogenous protein is below the detection limit of our antibodies. We demonstrate that hNEF-sp is a 3′ → 5′ exoribonuclease that is active on ssRNA substrates and likely functions in the nucleolar compartment. The importance of the two RNA-recognition motifs (RRMs) in the protein was not examined in this study, but these may perhaps help identify certain RNA substrates. Alternatively, potential protein complexes formed by the enzyme might help select the RNA targets. However, in vitro nuclease assays used RNAs with a variety of sequences and these were all efficiently processed.

Human NEF-sp is a highly conserved protein, with orthologs having a similar domain architecture being detected in amphibians, reptiles, birds, and mammals. Although proteins bearing similarity within the region encompassing the nuclease domain of NEF-sp are detected in insects, they lack the two tandem RRMs. A similar situation exists in the budding yeast *Saccharomyces cerevisiae*, where RNA exonuclease 1 (Rex1p)/Rnh70p/yGR276 (van Hoof et al. 2000) bears similarity to the nuclease domain of NEF-sp. Rex1p is a nuclear protein that is shown to use its 3′ → 5′ exonuclease activity to trim the extended 3′ end of precursors of 5S rRNA, tRNA^Arg^, and tRNA^Met^ (van Hoof et al. 2000; Ozanick et al. 2009). 5S rRNA is transcribed by RNA polymerase III as precursors with a 3′ extension. Such extended 5S rRNA or tRNA precursors accumulate in the *REX1* mutant yeast (van Hoof et al. 2000) and also become polyadenylated by nuclear surveillance machinery (Ozanick et al. 2009). However, this maturation process mediated by Rex1p is not essential for viability. In fact, the presence of the 3′-extended 5S rRNA precursors do not seem to affect the maturation of rRNA in ribosomes (Nariai et al. 2005), as sufficient mature rRNA and tRNA molecules get made due to the action of a number of redundant nucleases (van Hoof et al. 2000; Ozanick et al. 2009).

Our own analysis of the *Nef-sp* mouse knockout mutant revealed no obvious phenotype. Testis transcriptome analysis did not reveal any dramatic change in RNA species, except for the *Nef-sp* transcript itself (Fig. 4G). Some repeat transcripts were up-regulated in the mutant, but failure to silence them seems to have no consequence for mouse fertility (Fig. 4H). It is possible that precise trimming of the unknown substrate(s) may not be important for viability/fertility due to potential complementation by other nucleases. Nevertheless, our study provides the first biochemical and genetic characterization of the mammalian NEF-sp exoribonuclease.

## MATERIALS AND METHODS

### Clones and constructs

The coding sequences for human (LOC8169) and mouse (2610020H08Rik; MGI:1919402) RNA exonuclease 5 (Rexo5) or *Nef-sp* were amplified by reverse transcription PCR (RT-PCR) from adult human testes total RNA (Clontech, cat no. 636533) or mouse testes RNA, respectively. GenBank accession numbers are human *NEF-sp* (NM_001199053), mouse *Nef-sp* long isoform (KY853396), and mouse short isoform (KY853397). For mammalian cell culture expression, the following coding sequences were cloned into the SalI and NotI sites of the vector pCI-neo-N-HA (Pillai et al. 2005): hNEF-sp (1–774 aa), hNEF-sp^ΔNLS^ (58–774 aa), mNEF-sp (1–784 aa), mNEF-sp^ΔNLS^ (53–784 aa), and mNEF-sp short isoform (1–696 aa).

### Cell culture and antibodies

Human epithelioid cervix carcinoma (HeLa) and mouse NIH/3T3 cell lines growing on coverslips in 12-well plates were transfected with 2.5 µg of expression plasmids using X-tremeGENE HP (DNA Transfection Reagent, Roche; 06366236001). Subcellular localization of HA-tagged proteins was examined using the anti-HA as primary antibody, followed with staining by secondary antibody anti-mouse-IgG coupled Alexa Fluor 488 (Invitrogen). Cells were examined under an inverted confocal microscope Leica TCS SP2 AOBS.

### Recombinant human NEF-sp production

For production of recombinant proteins using the eukaryotic expression systems we used the following ovary-derived insect cells: Sf21 from the Fall Army worm *Spodoptera frugiperda* or High Five (Hi5) from the cabbage looper, *Trichoplusia ni*. The full-length human NEF-sp (1–774 aa) or a mutant version (hNEF-sp^Mut^) carrying a point mutation (D313A) were cloned into the NheI and KpnI sites of the vector pACEBac2 (6xHis-Strep-SUMO-TEV-fusion) (Bieniossek et al. 2012). Proteins were purified by Ni^2+^-affinity chromatography in lysis buffer (25 mM Tris, pH 7.5, 500 mM NaCl, 5 mM of β-mercaptoethanol, 0.5% Tween, 20 mM imidazole, 5 mM MgCl_2_, 10% of glycerol) supplemented with protease inhibitor (Roche; Complete EDTA-free). The protein was further purified on a Heparin column (HI-Trap; GE Healthcare) and monodisperse fractions collected by gel filtration chromatography (25 mM Tris, pH 7.5, 500 mM NaCl, 5 mM of β-mercaptoethanol, 0.5% Tween, and 10% of glycerol) (Superdex 200; GE healthcare). Proteins were used in nuclease assays without removal of the tags.

### Nuclease assay

The ssRNA (Microsynth) and ssDNA (Invitrogen) substrates (see list below) were labeled at the 5′ end with [γ-^32^P]ATP (PerkinElmer; NEG002A001MC) and T4 polynucleotide kinase (Thermo Scientific; EK0031). The labeled probes were resolved in 15% denaturing urea–polyacrylamide gels, and full-length labeled RNAs were gel-eluted with 0.3 M NaCl followed by phenol–chloroform extraction, precipitation and resuspension in 20 µL of RNase-free water.

RNA and DNA duplexes were made as described: 100 µM of the top strand was labeled at the 5′ end with [γ-^32^P]ATP and resolved in 20% in denaturing urea–polyacrylamide gels. The full-length labeled oligonucleotides were eluted in 600 µL of elution buffer (300 mM NaOAc, 1 mM EDTA, and 0.5% SDS) overnight at 4°C followed by precipitation using 1.8 mL of 100% ethanol and 2 µL of glycogen. The pellets were resuspended in 16 µL of water and annealing reactions were performed using 2 µL of 100 µM unlabeled complementary strand and 2 µL of 10× duplex-annealing buffer (100 mM MOPS, pH 6.5, 10 mM EDTA, and 0.5 M KCl) followed by heating the samples to 95°C and gradually cooled down to room temperature. The oligonucleotide duplexes were resolved on 15% nondenaturing PAGE followed by gel elution and precipitation as described above.

We mixed 1 µL of RNA/DNA substrate with 1 µM of purified recombinant human NEF-sp proteins (wild-type or point mutant [D313A]) and incubated at 37°C for 1 h in buffer (25 mM Tris–HCl, pH 7.5, 150 mM NaCl, 2 mM DTT). Divalent metal ions needed for the reaction were provided by supplementing the reaction with 25 mM MgCl_2_ or 2.5 mM MnCl_2_. When required, 150 mM EDTA was added to chelate Mg^2+^ ions. Treatment with Proteinase K was used to terminate the reaction, followed by phenol–chloroform extraction of the RNA and precipitation with ethanol. The samples were resolved by electrophoresis on 15% denaturing urea–polyacrylamide gels and exposed overnight to a Phosphor Storage screen (GE Healthcare Life Sciences). Screens were scanned using a Typhoon Scanner (GE Healthcare Life Sciences). ssRNA1 was used for the nuclease assays of Figure 2, B and C. Figure 2D was made using ssRNA1, ssRNA2, and ssRNA3. The nuclease assay with 2-*O*-Met modification at the 3′ end was done using ssRNA2 (without modification) and ssRNA 2-*O*-Met_1 (Fig. 2E). dsRNA was obtained by annealing ssRNA4 and ssRNA5 (Fig. 2F), and dsDNA by annealing ssDNA1 and ssDNA2 (Fig. 2G). Hairpin 1 (without overhangs) and Hairpin 2 (with 10-nt 3′ overhang) were used for the nuclease assays of Figure 2H.

### Mouse mutant generation

The coding sequence in the *Nef-sp* locus of the mouse genome was disrupted using the RNA-guided Cas9 endonuclease (Cas9 nickase [Cas9n]) and a pair of guide RNAs (gRNAs) (Mouse Biology Program, University of California, Davis). The *Cas9n* mRNA and the in vitro transcribed gRNAs (gRNA L1 and R4) were used for injection into C57B1/6J host embryos for mouse generation using one-cell stage injection (Fig. 4B). The founder females carrying a 23-bp out-of-frame deletion were crossed with wild-type C57Bl/6J Rj males (Janvier Labs, France) to establish the mutant line.

#### Tail genomic DNA isolation and genotyping PCR conditions

Tails were digested in buffer (50 mM Tris–HCl, pH 8.0, 100 mM EDTA, 100 mM NaCl, 1% SDS with 25 µg of Proteinase K) at 55°C overnight. DNA was precipitated with isopropanol, washed in 70% (v/v) ethanol and resuspended in 10 mM Tris–HCl, pH 8.0. To identify the F1 animals we used a pair of primers, the forward primer (represented in blue) complementary to the region spanning the deleted region and the reverse primer around 500-bp downstream from the deletion (Fig. 4D). To distinguish between heterozygous and homozygous mice, an additional PCR was done using a forward primer that only anneals to the wild-type allele (represented in orange) (Fig. 4D).

Genotyping primers: SS323 (5′-GGTTTCATAACTTGACTGTG-3′), SS330 (5′-CAGATAGCAGTCCCCTCTTT-3′), and SS336 (5′-GGATTACGGAAACTCAAAGTG-3′). The PCR reactions were performed in 1× Taq Buffer (Fermentas), 200 µM dNTPs, 0.25 µM primers, 2.5 µM of MgCl_2_, 2 µL of DNA template, and 0.5 µL of Taq DNA polymerase in a final volume of 25 µL. PCR conditions were 95°C for 5 min, then 40 cycles of 30 sec at 95°C, 30 sec at 60°C and 30 sec at 72°C, and a final step at 72°C for 5 min.

### Total RNA libraries and bioinformatics

Total testicular RNA was isolated by TRIzol Reagent (Invitrogen cat. no. 15596026). Strand-specific RNA-seq libraries were prepared using the TruSeq Stranded Total RNA Sample Preparation Kit (Illumina) after removal of abundant ribosomal RNAs with Ribo-Zero (mouse). Libraries were sequenced with the Illumina HiSeq 2000 platform (EMBL Heidelberg Gene Core facility) for 50 cycles.

Reads were sorted into individual libraries based on the barcodes; the 3′ adapter sequences were removed and mapped to the mouse genome (mm9). The software used for processing the data (genomic coordinates, etc.) from the raw data files are in-house tools developed by the Sachidanandam laboratory (Olson et al. 2008). Only reads perfectly matching the genome were kept for further analysis. The reads were divided into groups based on their annotation (repeat sense, repeat antisense, gene exon sense, gene exon antisense, gene intron sense, gene intron antisense, ncRNA sense, ncRNA antisense, miRNA sense, miRNA antisense, none) and their counts were compared between *Nef-sp*^−/−^ and *Nef-sp*^+/−^. DESeq2 bioconductor package (Love et al. 2014) was used to search for differentially expressed transcripts. An adjusted *P*-value of 0.05 was used as a threshold for statistical significance.

RNA secondary structures were predicted using the Mfold web server (Zuker 2003).

## DATA DEPOSITION

Deep sequencing data sets generated in this study are deposited with the Gene Expression Omnibus (GEO) under accession number GSE97235.
